# Combating Health Care Fraud and Abuse: Conceptualization and Prototyping Study of a Blockchain Antifraud Framework

**DOI:** 10.2196/18623

**Published:** 2020-09-10

**Authors:** Tim Ken Mackey, Ken Miyachi, Danny Fung, Samson Qian, James Short

**Affiliations:** 1 UC San Diego - School of Medicine Department of Anesthesiology and Division of Infectious Diseases and Global Public Health La Jolla, CA United States; 2 San Diego Supercomputer Center BlockLAB La Jolla, CA United States; 3 Global Health Policy and Data Institute San Diego, CA United States; 4 UC San Diego - Extension Department of Healthcare Research and Policy La Jolla, CA United States; 5 LedgerSafe Corporation San Diego, CA United States; 6 Institute of Electrical and Electronics Engineers San Diego, CA United States

**Keywords:** fraud, blockchain, medical informatics, delivery of healthcare, Medicare, information science

## Abstract

**Background:**

An estimated US $2.6 billion loss is attributed to health care fraud and abuse. With traditional health care claims verification and reimbursement, the health care provider submits a claim after rendering services to a patient, which is then verified and reimbursed by the payer. However, this process leaves out a critical stakeholder: the patient for whom the services are actually rendered. This lack of patient participation introduces a risk of fraud and abuse. Blockchain technology enables secure data management with transparency, which could mitigate this risk of health care fraud and abuse.

**Objective:**

The aim of this study is to develop a framework using blockchain to record claims data and transactions in an immutable format and to enable the patient to act as a validating node to help detect and prevent health care fraud and abuse.

**Methods:**

We developed a health care fraud and abuse blockchain technical framework and prototype using key blockchain tools and application layers including consensus algorithms, smart contracts, tokens, and governance based on digital identity on the Ethereum platform (Ethereum Foundation).

**Results:**

Our technical framework maps to the claims adjudication process and focuses on Medicare claims, with the US Centers for Medicare and Medicaid Services (CMS) as the central authority. A prototype of the framework system was developed using the blockchain platform Ethereum (Ethereum Foundation), with its design features, workflow, smart contract functions, system architecture, and software implementation outlined. The software stack used to build the system consisted of a front-end user interface framework, a back-end processing server, and a blockchain network. React was used for the user interface framework, and NodeJS and an Express server were used for the back-end processing server; Solidity was the smart contract language used to interact with a local Ethereum blockchain network.

**Conclusions:**

The proposed framework and the initial prototype have the potential to improve the health care claims process by using blockchain technology for secure data storage and consensus mechanisms, which make the claims adjudication process more patient-centric for the purposes of identifying and preventing health care fraud and abuse. Future work will focus on the use of synthetic or historic CMS claims data to assess the real-world viability of the framework.

## Introduction

### Background

Fraud and abuse is a major financial, legal, and policy challenge in the US $3.5 trillion United States health care system, with the Department of Justice (DOJ) and the Department of Health and Human Services (HHS) reporting recoveries estimated at US $2.6 billion in the fiscal year 2019 alone [1]. In fact, recoveries for health care fraud and abuse have steadily risen in the past 5 years, with settlements consistently exceeding US $2 billion over the past decade [2]. In 2018, the DOJ announced the largest national health care fraud takedown in history, which included over 601 defendants charged across 58 federal districts and involved 165 health care professionals, equating to a total of US $2 billion in false billings, including illegal distribution of opioids and narcotics [3].

Health care fraud and abuse involve all sectors of the health care industry, including drug and device manufacturers, hospitals, pharmacies, physicians, wholesalers, distributors, laboratories, and payers. Arguably, the most significantly impacted group is payers, including public agencies such as Medicare, Medicaid, and Tricare as well as private payers, who are defrauded of billions in health care claims yearly [4,5]. Fraudulent health care occurs in different forms, including kickbacks, false claims (eg, billing for services not rendered, upcoding, and provisioning of medically unnecessary services), and illegal self-referrals [5,6]. Fraud and abuse have a direct negative impact on health care utilization as it leads to a waste of limited resources and potentially endangers patients by providing them unnecessary care or precluding their access to medically needed services, which can lead to a higher risk of all-cause mortality and emergency hospitalization [4,6].

Enforcement against health care fraud and abuse comes in the form of well-established legal mechanisms focused on penalizing such actions, including (1) the False Claims Act, United States Code (USC) section 3729 to 3733; (2) the Antikickback statute, 42 USC section 1320a to 7b(b); (3) the Physician Self-Referral Act (Stark law); (4) the Exclusion Statute; and (5) the Civil Monetary Penalty Law [5,7]. Prosecution for fraud and abuse can lead to civil (monetary) penalties (including triple damages) for each claim or service, and in some cases, criminal penalties, including possible exclusion from federal and state reimbursement programs. These legal frameworks serve as a strong deterrent to fraud and abuse schemes, but detection and prevention remains an ongoing challenge.

Although efforts have been made to automate the detection of fraud and abuse through computational methods involving data mining of Medicare and Medicaid reimbursement claims data sets, most of the fraud and abuse prosecutions continue to originate from whistleblowers [8-10]. Whistleblowers are incentivized to report fraud and abuse activities through *qui tam* provisions that allow private individuals acting as *realtors* to bring a suit on behalf of the government [5,11]. Once a lawsuit is filed, the DOJ then has the option to intervene and join one or all of the counts of a pending qui tam action. If the claim concludes in a prosecution and settlement, then the whistleblower may be entitled to 15% to 30% of what is recovered, a clear incentive for reporting, although *blowing the whistle* may come at a high personal and professional cost [5,11].

This current system of relying on whistleblowers to detect and report fraud and abuse is subject to certain challenges, including court cases, some of which limit the protection for prospective whistleblowers [5,12]. Furthermore, prosecutions based on whistleblowers’ reports are not always successful, are often skewed toward prosecution of higher-amount cases, and by nature are reactive and punitive rather than proactive in preventing fraud and abuse. Hence, new technology approaches are needed to enable better resilience, provenance, and verifiability of health care claims that may be susceptible to fraud and abuse, an activity that is aligned with antifraud and program integrity priorities currently being pursued by the US Centers for Medicare and Medicaid Services (CMS).

### Objective

One technology with the potential to address these challenges is blockchain, a distributed ledger technology with use cases across several industries, including the energy sector, transportation, finance, and health care [13]. Blockchain use cases in health care are beginning to mature, primarily to improve the governance of health care data and processes [14-16]. One of the primary uses involves improving management; enabling sharing; and improving exchange of patient health data, consumer health data, and genomic data [15,17-20]. This also extends to the use of blockchain for clinical research to improve trial data management and electronic consent [21]. Use cases in other health care sectors are also taking shape, including blockchain for pharmaceutical supply chain challenges (eg, detection of falsified medicines) and integration with medical devices and the internet of things [22,23]. Many of these uses focus on patient-centric approaches to manage and preserve the privacy of health care data with blockchain [24-27].

Importantly, incorporating blockchain into a systems software architecture can enable immutability, consensus, create incentives, and manage external data into a self-executing system with transparent rules across multiple stakeholders [14]. On the basis of these benefits, we proposed a technical framework for a blockchain-based system that includes 3 key stakeholder groups in the health care claims workflow process to enable a more proactive antifraud and abuse system. Although several companies are exploring blockchain to enhance health care reimbursement and revenue cycle management, few have explicitly assessed whether the technology can improve claims verification and better enable the detection and prevention of health care fraud and abuse [28,29]. Hence, this paper will explore the utility of blockchain by developing a fraud and abuse technical design framework and prototype built on the blockchain environment Ethereum.

## Methods

### Overview

Blockchain’s core utility is as a distributed database of transactions, securely connected in chronological order, enabling efficient and cost-reducing improvements to current systems and business processes [14]. Driving the efficiency of these blockchain processes is the enforcement and execution of rules by software, a shared governance environment, and use of smart contracts to create a more transparent and rule-based system that can tackle issues of trust, such as addressing fraud and abuse. Furthermore, health care information and communication technology systems are increasingly moving toward more *patient-centric* designs, not only for receiving the input from the patient but also for involving the patient in the design and solution implementation process [14,27,30].

Our blockchain technical framework leverages key principles of establishing trust through shared accountability and governance and enables the patient to be a stakeholder in addressing health care fraud and abuse. The primary aims of the blockchain solution are to (1) improve detection of potential fraudulent and illegal health care transactions and reimbursements, (2) create a more inclusive process for validating claims deploying a patient-centric approach, and (3) enhance efficiency in the claims adjudication process through smart contract automation.

To conceptualize this approach, we adopted the *fit-for-purpose* theoretical framework as published by Mackey et al [14] for designing health blockchain use cases that outline design and technical principles. These features are outlined below and are also described in the context of our early prototype version of the technical framework. On the basis of the central need for an environment that enables shared governance, we conceptualized our technical framework and prototype on the Ethereum decentralized platform, which enables 3 specific technical features that map to our use case, including (1) democratic autonomous organizations (DAOs), (2) smart contract execution environment, and (3) tokens via the ERC-20 token standard.

Hence, our design framework and prototype are based on the combination of *fit-for-purpose* design principles and feature layers on Ethereum.

### Ethics Approval and Consent to Participate

Ethics approval and consent to participate was not required for this study. All information collected from this study was from the public domain, and the study did not involve any interaction with the users. User indefinable information was removed from the study results.

## Results

### General Design

**Figure 1:**
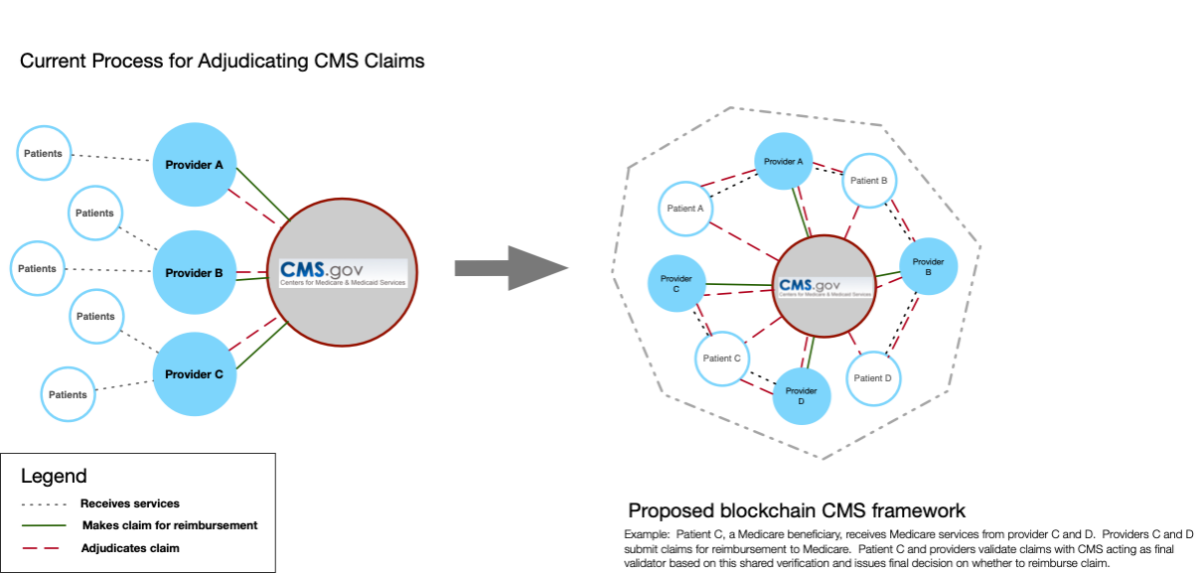
Visualization of the current process for Centers for Medicare and Medicaid Services claims reimbursement and proposed blockchain framework. CMS: Centers for Medicare and Medicaid Services.

The proposed solution to improve health care fraud and abuse detection will utilize a *hybrid* or consortium permissioned blockchain model that incorporates relevant stakeholders in the claims and reimbursement workflow, a transactional process that currently does not involve the patient (Figure 1). A consortium blockchain consists of both *public* and *private* blockchain characteristics by restricting participation to certain trusted users who act as nodes on the blockchain and are required to meet the criteria set forth by the consortium.

Importantly, a consortium-based blockchain model enables a high throughput of information to be validated and stored on the blockchain. This is due to the restricted number of validation nodes that both process and distribute information throughout the system. In contrast, the Bitcoin blockchain can process approximately 8 transactions per second, and the public Ethereum blockchain can only process approximately 15 transactions per second, partly owing to the massive number of nodes connected to their respective networks. This is not sufficient for a health care fraud detection system, which requires significantly more than 100 transactions per second. The consortium blockchain model enables the system to scale to process large number of transactions while restricting participation and enhancing the security of entities who can access and interact with the system.

Our technical framework includes all subgroups (eg, providers, payers, and patients) that are eligible to provide or receive services through Medicare. We focus on Medicare as it is the largest public payer system in the United States (with projections that Medicare expenditures will increase from US $705 billion in 2017 to US $1.436 billion in 2027) and as many of the legal frameworks associated with health care fraud and abuse only apply to public sector reimbursement, although states may have their own laws and regulations when it comes to the private pay or the employer insurance–based market [31]. We primarily focus on Medicare claims under Parts A and B, but not C (Medicare Advantage), as these are capitated payments.

Next, we discuss the framework’s core design features of shared data governance, interoperability, the smart contract claims validation process, proposed system architecture, and privacy considerations.

### Shared Data Governance

The first step in setting up a shared governance system is to define the membership of the distributed community that will participate in the consortium technical framework. A DAO will govern the framework with its subgroups of providers, payers, and patients based on verification that they are eligible to provide and receive Medicare services. Validating membership in these DAO subgroups will be accomplished by cross-referencing information directly from CMS Medicare identifier data (see Multimedia Appendix 1 [14] for more details on DAO subgroup matching). This will include matching CMS information on eligible providers (eg, clinicians accepting Medicare-approved payments), eligible provider organizations (eg, health care facilities accepting Medicare patients and clearinghouse organizations authorized to bill on their behalf), and eligible Medicare beneficiaries (eg, patients).

Furthermore, although blockchain is generally described as a decentralized network, this use case relies on a central payer to make a final adjudication decision on claims. Hence, the central authority of this consortium blockchain will be CMS, although the process of claim validation will be shared by the DAO. In this role, CMS will have privileges to add providers, gain access to universal claim information from providers and their associated patients, and adjudicate claims based on CMS’ own set of rules and regulations that meet specific statutory criteria to validate that a service was actually billed and received by the patient appropriately. The framework also allows CMS to delegate and determine participation permission and access to different subgroups and determine validation rules in the network. For example, CMS may determine that there must be complete agreement among the associated patient, provider, and CMS for a claim to be confirmed and validated.

The DAO participants will act as *authority* nodes, which will validate the claims data that are proposed to be written to the blockchain. Importantly, the permissions structure for the validation procedure will be claims and patient-specific, with only the relevant provider, organization and beneficiary gaining access to identifiable reimbursement claims data, subject to identity verification. Data join fields (when contents of one database or table are joined based on a common attribute field), including type of payment (eg, fee-for-service and prospective payment systems), medical billing codes (eg, International Statistical Classification of Diseases and Related Health Problems-ICD-10, Current Procedural Terminology-CPT, Diagnosis Related Group-DRG, and Healthcare Common Procedure Coding System-HCPCS level 1 and 2 codes), and National Provider Identifier and patient’s Medicare ID number of Medicare Beneficiary Identifier, can be used to validate identity and permissions for claims verification processes.

**Figure 2:**
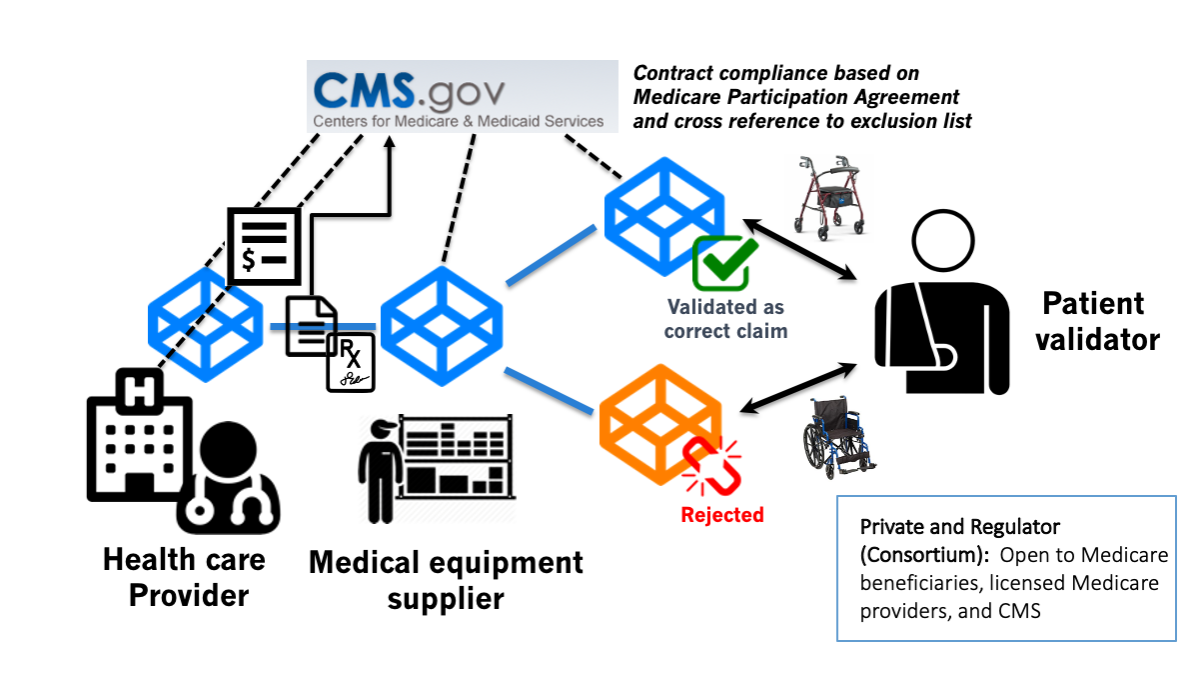
An example of a claim for a durable medical equipment, where a health care provider submits a claim for a walker that is supplied by a durable medical equipment provider, with the identity of the provider, durable medical equipment provider, and patient beneficiary validated by CMS. The claim is then either validated or rejected based on whether the patient actually receives the correct medical equipment. In this example, CMS was billed for a wheelchair, but the patient received a walker and validated that the claim was incorrect, leading to a potential denial of claim and detection of a false claim. CMS: Centers for Medicare and Medicaid Services.

This validation of participating nodes will help prevent fraudulent actors from submitting claims (including *ghost* patients, ie, patients who do not exist or never received services, and providers and organizations debarred from Medicare participation). An example of how this would apply to a medical equipment claim is provided in Figure 2.

### Interoperability and System Integration

Key to the functioning of processes to validate participating nodes and health care claims is establishing the interoperability of the system to interact with off-chain databases and systems, including CMS databases of verified Medicare providers and beneficiaries, medical coding and billing systems (including front-end and back-end billing and health care electronic data interchange), revenue cycle management systems, and current Medicare billing and antifraud solutions. Integration with CMS databases of validated providers and beneficiaries, either through a data clearinghouse model (ie, not directly integrated with CMS systems but using a third-party data clearinghouse) or through existing application programming interface (APIs; with query function such as the CMS Data portal API in JavaScript Object Notation-JSON) to directly query CMS or provider databases, will be a priority. In addition, integration with new CMS patient-centered initiatives, such as the Medicare’s Blue Button 2.0 (explained later in the *Discussion* section), will also be explored.

**Figure 3:**
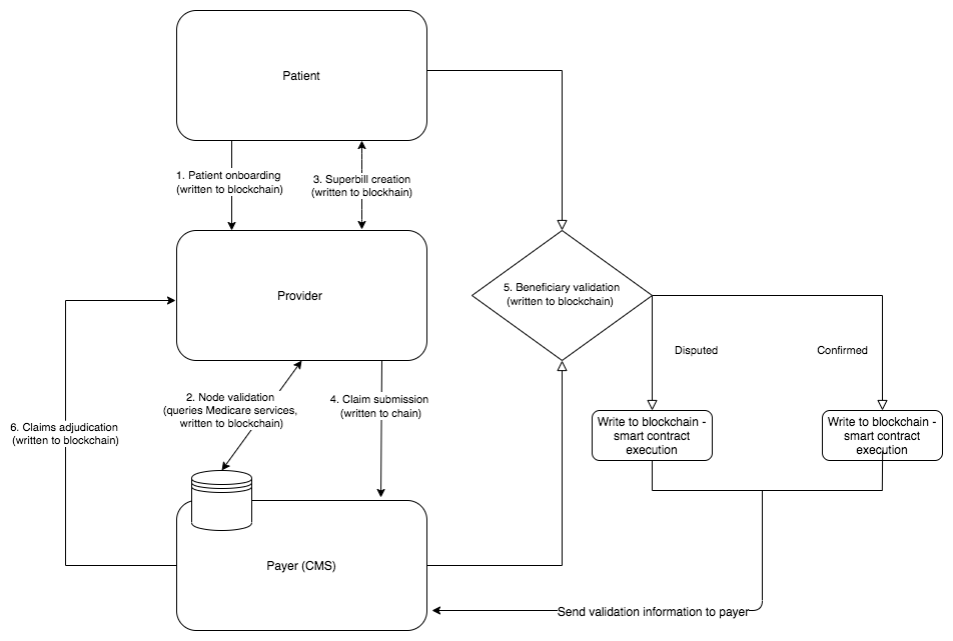
Process for validating data off-chain in proposed framework. CMS: Centers for Medicare and Medicaid Services.

To better ensure broader health informatics interoperability, our technical framework is also intended to ingest data solely related to the electronic claims submission process for Medicare Part A and B using the Accredited Standards Committee X12 standard transmission format (also known as Health Insurance Portability and Accountability Act [HIPAA] 5010), although it could also integrate into electronic health records (EHRs) systems using the Health Level Seven International Fast Healthcare Interoperability Resources (HL7 FHIR) standard. Finally, to enable interoperability while maintaining privacy, a secure off-chain key-value store, which can only be accessed and modified through a trusted execution environment within the system, will map the address or identifiers of users in our framework to the CMS identifiers. Validation of correctness and integrity between an off-chain database and the blockchain network may be performed using 2 patterns known as the challenge response pattern and the off-chain signature pattern (Figure 3 and Multimedia Appendix 1) [32].

### Smart Contract Claims Validation Process

For the desired utility of our technical framework, it is necessary to map the Medicare claims submission, adjudication, and reimbursement processes to our framework’s data governance and smart contract feature layer. Below, we describe the basic claims workflow for the proposed framework that maps to the key procedures of verifying Medicare eligibility, creating a claim, submitting a claim, patient verification, and final adjudication. The proposed claims workflow, which will be automated by smart contracts in the framework, is described in Table 1.

Importantly, at each step of the Medicare claims adjudication process, smart contracts will govern how information is shared, what data are required, and how consensus is established for what is written to the blockchain. For consensus of validating and writing to the blockchain, our framework utilizes a proof-of-authority (POA) consensus mechanism. POA, a modified version of proof of stake, uses the validator’s identity as a form of stake to validate blocks to be written to the blockchain. In this sense, consensus of decisions on validating a claim for the network (eg, submission of claim, viewing claim, and final validation of claim) will be visible to all stakeholders involved in the claim (ie, the provider who rendered services, the patient who received the services, and CMS which acts as the payer for services) and will also be tied to the user’s identity. POA validation nodes should have their identity validated with CMS (as explained above) in the DAO, and their validation privileges can be revoked in cases of fraudulent behavior or if they are disbarred from Medicare. The rules of validation will be determined by the DAO to ensure that all stakeholders are accounted for. For example, the DAO may determine that over 90% of all authority nodes must validate information before being written to the blockchain.

As the central authority on our framework, CMS will set the rules for smart contracts, the parameters of consensus at each step, and the permission and data governance structure for the network, subject to federal laws such as 42 USC section 1395 and the following, 42 Code of Federal Regulations section 400 and the following, and HIPAA. The provider will have access to the framework and smart contract layer to add patients, provide services, and file claims based on the services they have rendered and where they have submitted a claim on behalf of a Medicare beneficiary. Patients will have access to view the services and associated claims that have been submitted with their digital beneficiary identity (eg, Medicare number) and have a role in validating those claims.

**Table 1:** Description of mapping the framework’s smart contract processes to the Centers for Medicare and Medicaid Services general claims adjudication process.

Step	Claims process	Description
1	Patient onboarding	Patient registers and onboards at the provider location, confirms Medicare eligibility, and schedules an appointment—written to chain
2	Node validation	Patient, provider, and/or organization is validated for eligibility for Medicare services and benefits—query network and written to chain
3	Superbill creation	Medicare eligible services are provided to the patient by a health care provider and organization and a “superbill” (comprising claim codes and patient information) is created—written to chain
4	Claims submission	Provider submits claim directly to CMS^a^ or uses a third party (ie, clearinghouse)—written to chain
5	Beneficiary validation	Patient beneficiary to the claim is queried to validate the services received upon a filed claim—written to chain
6	Claims adjudication	Payer (CMS) adjudicates the claims with validation information from both the provider and patient records and executes proof-of-authority consensus across other validating nodes (ie, patient and provider)—consensus results—written to chain
7	Electronic remit advice form	Payer (CMS) assesses whether to accept, deny, or reject a claim and provides payment information via an electronic remit advice form—written to chain

^a^CMS: Centers for Medicare and Medicaid Services.

### Prototype System Architecture

Our technical framework consists of a web application (front end), blockchain network, and off-chain database storage (back end). The web application will display the system information on a graphical user interface (GUI). The blockchain network will validate and record all transactions that occur in the system. The off-chain database storage will store information regarding user credentials as validated by CMS as well as protected health information (PHI) or personally identifiable information (PII) not stored on the blockchain network but already present in existing clinical and EHR systems.

The system will implement a hybrid consortium blockchain model, wherein authorization is required to join the network. POA consensus will enable validation power among different stakeholders in the system in a distributed manner to mitigate collusion and false records among patient and provider workflows. Different read and write permissions will be given to different stakeholders in the network depending on the smart contract claims adjudication processes previously outlined. All transactions will be indexed in a system administration database to audit and enable efficient queries of aggregated data from the entirety of the network.

**Figure 4:**
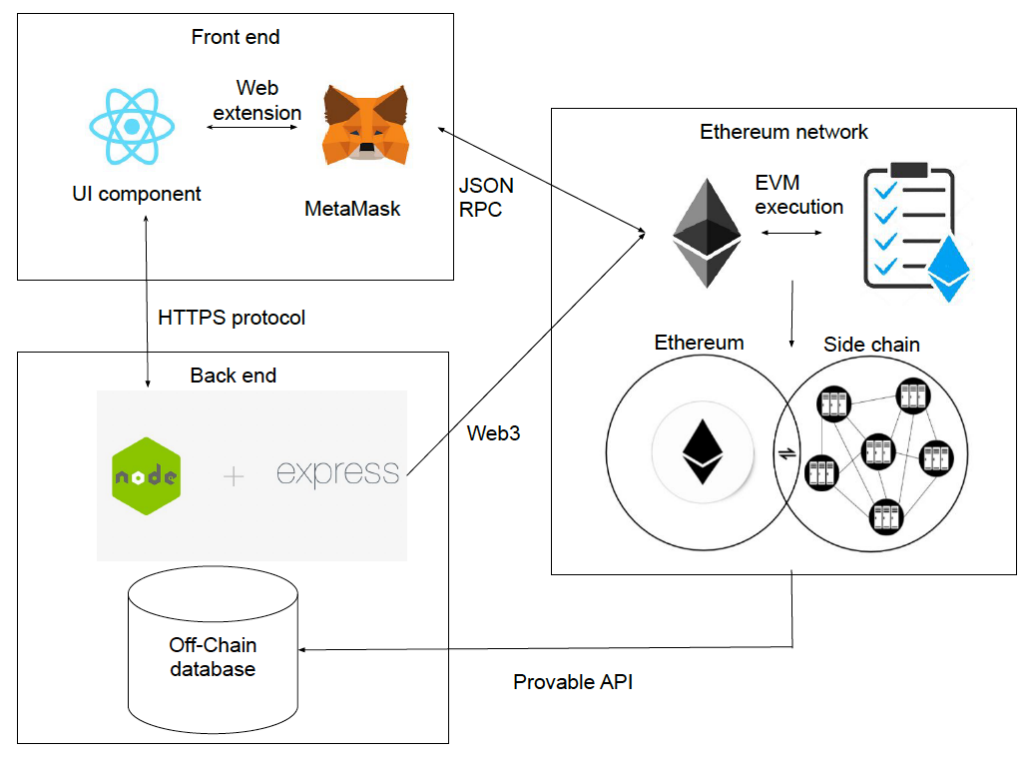
Overall system architecture of the framework, with React used for the user interface and NodeJS and an Express server used for the back-end processing server. Solidity was the smart contract language used to interact with a local Ethereum blockchain network. The Application Programming Interface is a set of functions and procedures allowing communication between the front-end user interface, back end server, blockchain network as well as access to functions and data of the system. The Ethereum Virtual Machine is the runtime environment for smart contracts in Ethereum. JavaScript Object Notation remote procedure protocol is a specification that defines several data structures and the rules around their processing. Interaction with the Ethereum blockchain starts with sending a request via JSON RPC. API: Application Programming Interface; EVM: Ethereum Virtual Machine; JSON: JavaScript Object Notation; RPC: remote procedure protocol.

A prototype of this blockchain Medicare fraud and abuse framework can be explored and run by downloading and following instructions from our GitHub Repository (San Diego Supercomputer Center-BlockLAB Medicare-Claim- Verification). The software stack used to build the system consists of a front-end user interface, a back-end processing server, and a blockchain network (Figure 4). The blockchain application can be executed by running a local Ethereum blockchain via Ganache; deploying smart contracts onto the Ethereum blockchain via Truffle; installing all NodeJS packages; and running the NodeJS application, which is connected to the Ethereum network through Web3.

**Figure 5:**
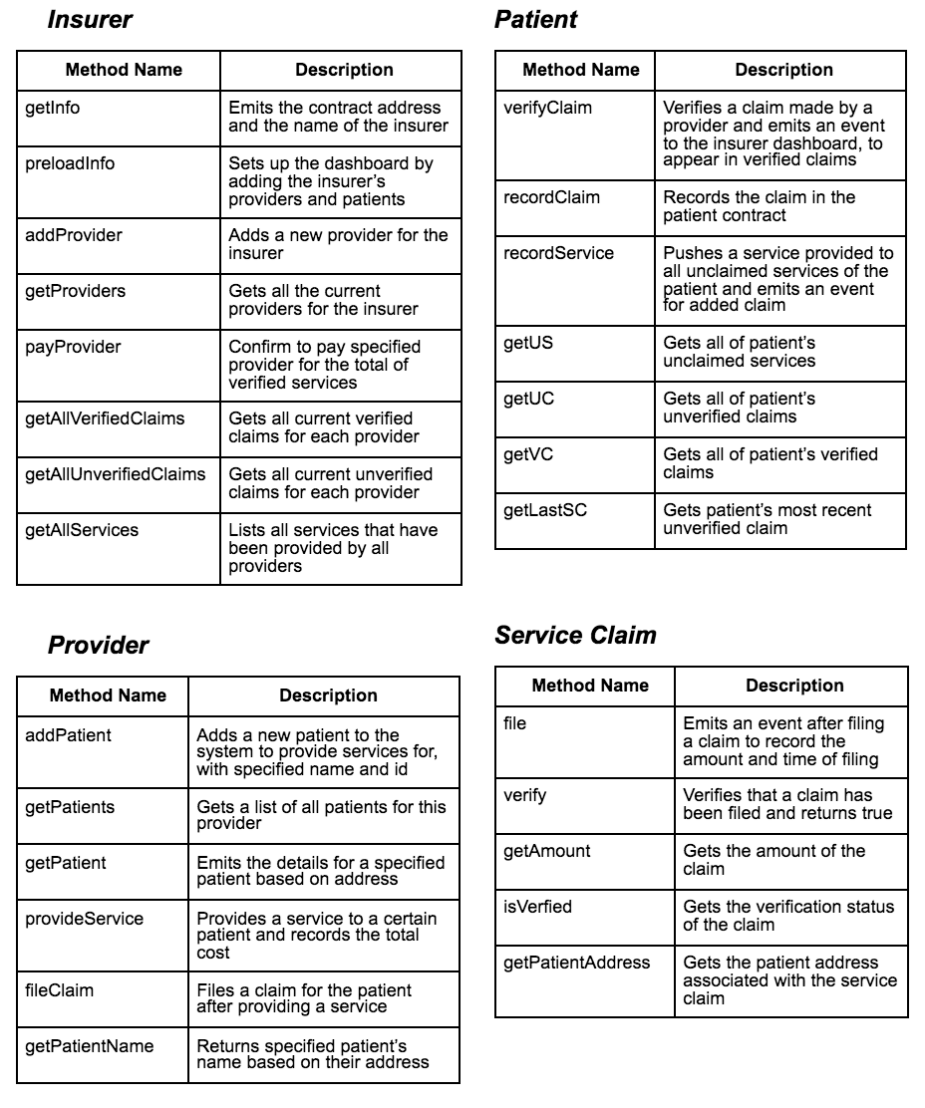
Description of the framework’s smart contract function inputs.

The front end of the web application will comprise 3 separate GUIs corresponding to the different roles in the system (eg, payer, provider, and patient) and will map directly to the smart contract claims adjudication function inputs (Figure 5). The roles of the system will be determined by a registration process in which users’ credentials will be validated against CMS registries, confirming proper identification and roles in the Medicare reimbursement process. For example, a patient of the system must provide their Medicare beneficiary number, which will be cross-referenced off-chain against the Medicare database to validate access to the system. Upon proper registration, the user credentials will be stored as well as their associated role, which will be validated with input information from a log-in GUI.

The payer (ie, CMS) GUI presents information about the providers and claims. The current implementation breaks down the claims into 2 lists, verified claims and unverified claims, for the payer to distinguish whether a patient has verified a Medicare claim submitted. The provider GUI presents information regarding patients and allows providers to write information to the blockchain regarding both providing a service and filing a corresponding Medicare claim. The patient GUI presents information regarding the current filed claims made on their behalf and allows a patient to confirm or dispute whether they were provided the health care service or benefit associated with the claim.

Users designated with the payer role also have access to onboard providers who have been verified and registered in the system, pay and/or adjudicate a claim, and read all the claim information regarding providers and patients that have been registered and validated with their organization or agency (in our case, CMS). Users designated with the provider role have access to onboard verified patients and have access to read patient information that has been registered with their entity only. Users designated with the patient role only have access to confirm or dispute claims associated with their verified Medicare digital identity in the system.

The data storage of the current implementation can be categorized into on-chain storage, which is data written to the blockchain, and off-chain storage, which is data stored in a traditional database (structured query language-SQL or not only SQL-NoSQL) external to the blockchain network. On-chain data comprise entity relationship information, such as which payer-provider relationships and provider-patient relationships need to be validated as part of the claim adjudication process. On-chain data also comprise information regarding the adjudication of services provided and claims submitted. The on-chain information is stored on Solidity smart contracts, which are deployed to a private Ethereum blockchain and are used to populate the GUIs of the web application via event listeners and Solidity contract calls (Multimedia Appendix 1). Off-chain data comprise user credential information, PHI, Medicare data, and other information needed to authenticate and integrate into the Medicare claims adjudication workflow, as previously discussed.

Finally, patient verification is a crucial step to adjudicate Medicare claims under our framework. To further incentivize patients to participate in the blockchain-based validation process, we have implemented an ERC-20 token to encourage active patient-generated claims validation (see Multimedia Appendix 1 for details on ERC-20 tokens). The ERC-20 token is meant to have dual utility within our framework to encourage patient validation of claims and to incentivize other population health benefits. Further research will explore different utilities for the ERC-20 token to maximize patient participation, such as allowing use of tokens to lower patient cost sharing (ie, co-pays) and incentivizing other health behaviors (eg, issued tokens for claims validation used to lower the cost of prescription drugs and for fitness club memberships).

### Privacy Considerations

As patient claims data are subject to privacy and confidentiality under HIPAA, no PHI or PII will be written to the public Ethereum blockchain. Instead, access to all patient-level claims data will be restricted to permissions validated to the identity of providers and organizations of the DAO with specified roles involved in the claim (ie, HIPAA covered entities); any organization subject to a HIPAA business associate agreement, which needs access to such data; and the patient who is the sole beneficiary of the claim.

All PHI will be stored off-chain isolated in existing systems with identified join and match data attribute fields the only event queried (such as Medicare claim number or Medicare Beneficiary Identifier). The information that will be written to the chain is simply the adjudication of the claim information itself, with encrypted data regarding any patient identifiers, wherein management of public and private keys to access third-party databases will be needed to correlate any claim information with PHI or PII. Properly deidentified claims and associated metadata can be written to a separate public chain of the framework for inspection by all stakeholders, including regulators and law enforcement, which can use these data to detect larger patterns of fraud and abuse and suspicious claims or reimbursement activities through existing data mining and predictive analytics approaches.

## Discussion

### Principal Findings

The purpose of our blockchain fraud and abuse technical framework and prototype is to enable a shared governance approach to addressing health care fraud and abuse, while also empowering patients with the option and authority to become active participants in the claims verification process. We base this central design principle on 2 key facts: (1) for certain health care claims, patients are best suited to verify whether appropriate health care services have actually been rendered, and (2) patient verification or lack of verification of claims provides an important indicator of potential fraud and abuse risk, which can later be confirmed by investigation and can also enable targeted fraud and abuse prevention that is more proactive than current approaches. Hence, our framework focuses on a patient-centered design to address health care fraud and abuse by engaging the patient as a key stakeholder in the claims verification process.

We chose blockchain over existing technologies, such as cloud computing or traditional database storage methods, as our focus is on *shared* validation of claims in a distributed and immutable ledger that is supported by cryptography. Central to this model is establishing *trust* in a shared governance approach across multiple parties in the same transaction that also enables the patient to be an additional validating node in this process. These core features, along with the technology application layers enabled by blockchain (eg, smart contracts, digital identity, tokens, and consensus mechanisms), are why blockchain architecture is ideal for this use case.

Specifically, blockchain improves both the security and data integrity of the information stored in a system. However, blockchain technology is not suited for all systems as a data storage solution. To determine the viability of blockchain technology for a health care fraud detection system, we used a structured methodology and flowchart published by Wust and Gervais [33] to determine whether a blockchain is the appropriate technical solution*.* Properties such as public verifiability, transparency, privacy, integrity, redundancy, and the anchor of trust are all considered when determining whether a blockchain is a viable technology for a given problem. The decision flowchart applied to our fraud and abuse framework is available in Multimedia Appendix 1. By applying this methodology, we concluded that a consortium permissioned blockchain is an appropriate technical solution to solve the problems posed to current health care fraud detection systems.

Furthermore, the use of smart contracts on the blockchain enables an agreed-upon rule set to be automatically executed based on events. This automated event-driven architecture can also be accomplished by traditional systems, but these are generally controlled by a centralized entity. Health care insurance systems involve multiple stakeholders with different incentives. Stakeholders are often forced to trust an entity based on perceived compliance with applicable regulations. Trust in the health care claims system could be significantly improved by automating the execution of rule-based logic through smart contracts. For example, a specific action can be automatically invoked if a patient disputes a health care claim in our system, whereas current systems require detection and auditing of fraud mostly retrospectively.

Our framework also focuses on a patient-centric design, which is compatible with government-wide initiatives led by CMS, including the MyHealthEData initiative (which aims to provide patients with more access and control of their health care records from a device or mobile app of their choice) and the Medicare’s Blue Button 2.0 (which enables patients to access and share claims data) [34]. Importantly, Medicare’s Blue Button initiative provides Medicare beneficiaries with claims data in a universal and secure format, which can be integrated into our proposed framework. Although Blue Button 2.0 provides access to claims data to providers, it does not provide methods for beneficiaries to validate claims or report possible fraud and abuse directly to CMS mediated by technology. Hence, our patient-centric blockchain framework complements this and other CMS data access initiatives, while also ensuring that patients are not just consumers of their claims data but can also take action in the event of a discrepancy.

Central to our approach is also the fact that health care fraud detection has traditionally been implemented via reactive systems that analyze fraudulent claims activity after a claim has already been submitted and has likely been paid. These traditional systems can be costly (as defrauded amount may never be recovered or requires lengthy litigation) and rarely have incorporated any patient feedback when validating the integrity of a Medicare claim. Here, blockchain technology offers a potential solution by creating a tamper-evident and near-immutable audit log of health care claims and transaction data that can be viewed and agreed upon in a distributed ledger by providers, payers (eg, CMS), and patients to collectively verify claims and work collaboratively to identify fraud and abuse.

Our framework also aligns with specific programmatic priorities of HHS to combat health care fraud and abuse, including initiatives to reduce fraud, waste, and improper payments across its different agencies. The Affordable Care Act (ACA) has provided resources to CMS to improve prevention of fraud, waste, and improper payments through its CMS Fraud Prevention Initiative and Fraud Prevention toolkit that enables enhanced collaboration with state and law enforcement partners using predictive modeling technology. Furthermore, the ACA has empowered CMS to jointly develop many Medicare, Medicaid, and Children’s Health Insurance Program antifraud policies, leading to enhanced screening requirements for new providers and suppliers, a concept that aligns well with our stakeholder blockchain validation approach [35,36]. The overall objective of these approaches is to enable health care programs to do less *paying-and-chasing* of fraudulent claims and do more proactive and transparent fraud prevention [35].

CMS also has plans to develop a preventative model that will help identify potential fraud before it occurs by utilizing analytical techniques to improve payment accuracy by identifying, in real time, atypical trends that could be indicators of waste or fraud to appropriately intervene, again representing a good use case for patient validation data that can improve the precision of the proposed analytical models [35]. The rules provide new CMS enforcement tools to fight fraud, such as the ability to suspend payments in cases of credible allegations of fraud that could arise from a patient, and requires a more rigorous screening process for providers and suppliers enrolled in Medicare, including possible cross-termination for federal and state health programs [35]. Using these tools, Medicare and state agencies will be watching for trends that may indicate significant potential for health care fraud and can temporarily stop enrollment of a category or geographic area of providers or suppliers that has been identified as high risk.

Finally, HHS has been given new authority to prevent problematic providers from participating in Medicare. Specifically, the ACA increased the federal sentencing guidelines related to health care frauds involving US $1 million or more in losses to federal health care programs to create more disincentives for this activity. With this new authority also comes the responsibility of both determining and proving that a health care fraud has occurred. Hence, the collection of current CMS’ antifraud goals, initiatives, and authorities provides an opportunity to develop a blockchain-based Medicare fraud detection system that aligns with these objectives for the purposes of integrating and developing a fraud and abuse prevention model modernized for today’s health care and technology offerings.

### Possible Benefits, Limitations, and Challenges

The potential benefits of our framework focus on creating verified claims transaction logs and more efficient and validated workflows. First, instead of waiting for fraud and abuse to occur and then reacting to it retrospectively, the system will be designed to actively detect and prevent potential fraud and abuse and other noncriminal activities (eg, overbilling, unintentional upcoding, and billing errors) using a layer of patient validation that is not currently available in legacy claims adjudication systems.

It will also add a layer of aggregated data to detect more systemic forms of fraud and abuse that can be mined for geographic areas, vulnerable patient populations, and specific health care providers that may be prone to fraud and abuse activities. This claims workflow data generated by the framework, which can also be properly deidentified, could be written to a public chain for the purpose of data mining and research. In addition, cryptographically validated multistakeholder claims data (eg, the claim submitted, validating the identities of stakeholders, and consensus established about the claim) could also enable more efficient machine learning approaches to detect patterns and risk factors of fraud and abuse not available from current static claims data.

If our approach was implemented to augment the current health care fraud detection system and was able to prevent just 1% of the current lost value, it would have saved the US health care system over US $25 million. Furthermore, by automating a larger portion of the health care fraud detection system, cumbersome and tedious tasks such as the human review of health care claims could be reduced. Hence, incorporating a trust-based technology such as blockchain into health care fraud detection systems can have economic benefits and technical utility but needs further testing with real-world or synthetic data to assess feasibility.

However, there are also certain limitations and challenges associated with implementing our proposed Medicare blockchain fraud and abuse prevention system. First, full participation from all stakeholders in the Medicare claims lifecycle (eg, CMS, providers, and patients) will require a comprehensive process of integrating with existing information technology systems, identification of interoperability challenges, and ensuring the use of appropriate data standards (such as Blue Button 2.0, Accredited Standards Committee X12, and HL7 FHIR). However, integrating provider billing and revenue management cycle systems, existing Medicare databases and APIs, and a front end that can interact with the patient will likely prove challenging.

Furthermore, abuse of the framework system itself must be considered and mitigated. For example, there may be an attempt to manipulate the consensus between all stakeholders regarding a record of events. This includes situations where the beneficiary may be complicit with a fraud and abuse scheme, a situation where patient validation may actually lead to incorrect adjudication of a fraudulent claim. This is of specific concern when a patient may have a clear incentive to participate and benefit from fraud and abuse, such as in the context of opioid use disorder and drug diversion [37]. Our system will take multiple measures to identify and proactively prevent cases where both the provider and patient cooperate in fraud and abuse, with modifications to the smart contract claims adjudication process and consensus mechanism specific to high-risk claims and patient profiles. However, adding the patient to validation may also enhance anomaly detection when unusual validation behavior occurs and could also act as a cryptographically hashed affirmation and evidence of wrongdoing by a provider or patient for use in prosecution.

Owing to the different incentives available to providers, payers, and patients, a proper and mutually agreed upon consensus algorithm will need to be implemented to address many of these challenges. In addition, we have discussed the use of ERC-20 tokens in our framework to incentivize patients to verify claims. Along with ensuring that claims data are represented in a way that patients can understand and verify (including the translation of claims codes in lay terms and education on health literacy), proper incentives need to be in place to encourage patients to validate claims correctly. Tokens may also disincentivize bad behavior. For example, if a patient is complicit in a fraud and abuse scheme, token payments can be withheld, and even possible additional penalties could be applied to a patient’s validated Medicare identity. Hence, the *tokenomics* of reporting fraud and abuse and the benefits to the patient and Medicare itself, need further design and testing.

The framework we developed builds upon an emerging body of innovation seeking to transform the health care data and claims workflows using blockchain technology. However, all these proposals, including our own framework, face barriers to adoption and implementation that require further experimentation, assessment, and active collaboration with the health care community. In fact, although our framework focuses on CMS and Medicare, a similar consortium blockchain design tailored to a private payer’s own closed network of providers and beneficiaries might represent a more pragmatic approach to detecting fraud and abuse and enable better integration with more centralized systems. Future work on our framework will focus on reference models for different payer and provider network consortium types.

### Conclusions

Our blockchain framework proposes a tamper-evident and near-immutable audit log of health care claims and transaction data that can be viewed and agreed upon in a distributed ledger by providers, health care organizations, payers, regulators, and most importantly patients to verify claims for the purposes of limiting the loss of more than US $2 billion to health care fraud and abuse every year. Future studies of our proposed framework and prototype will need to focus on using synthetic or historic CMS claims data to assess the real-world viability of the framework.

## Appendix

Multimedia Appendix 1 Details on technical framework.

## Abbreviations

ACA: Affordable Care Act
API: application programming interface
CMS: Centers for Medicare and Medicaid Services
DAO: democratic autonomous organization
DOJ: Department of Justice
EHR: electronic health record
GUI: graphical user interface
HHS: Health and Human Services
HIPAA: Health Insurance Portability and Accountability Act
HL7 FHIR: Health Level Seven International Fast Healthcare Interoperability Resources
PHI: protected health information
PII: personally identifiable information
POA: proof-of-authority
SQL: structured query language
USC: United States Code
