# Supplementary material for: Combating Health Care Fraud and Abuse: Conceptualization and Prototyping Study of a Blockchain Antifraud Framework
Source: J Med Internet Res. 2020 Sep 10;22(9):e18623. doi: 10.2196/18623 (PMC7516680; doi:10.2196/18623)
Supplement: Multimedia Appendix 1 [file jmir_v22i9e18623_app1.docx]

**Supplementary Appendix 1: Details on Technical Framework**

**BASIC DESIGN PRINCIPLES:**

Key design principles for this framework are adopted based on the “Fit-for-Purpose” Framework (Mackey et. Al., 2019) that includes:

1. deciding on overall design features (e.g. public, private, and consortium blockchain designs);
2. setting rules for data governance (where data resides, who has permissions to data, and discussion about on-chain and off-chain storage);
3. exploring technical components (blockchain process workflow, web front end, consensus mechanism, and data exchange); and
4. the use of application layers (tokens, smart contracts, and digital identity).

**SHARED GOVERNANCE DETAILS:**

**DAO CMS sub-group matching:** This will include matching and verifying information on eligible providers who are validated as accepting Medicare-approved payments (matching provider data such as National Provider Identifier [NPI], specialty, and other data available on databases such as Medicare.gov Physician Compare). It will also include eligible provider organizations (e.g. hospitals, dialysis facilities, long-term care facilities, inpatient rehabilitation facilities, hospice care, home health services, and nursing homes) accepting Medicare patients and clearinghouse organizations who are authorized to bill on a provider’s behalf. The DAO will also include all eligible Medicare beneficiaries (matched based on their Medicare Beneficiary Identifier, Medicare Number, coverage data, and type of coverage).

**INTEROPERABILITY AND SYSTEM INTEGRATION:**

**Validation of off-chain sources and blockchain network:**

- 1. Challenge Response Pattern: The challenge response pattern utilizes an external state machine that receives information from the blockchain network through a smart contract regarding the user information (e.g. provider, patient) and performs computation to validate the associated information in an off-chain database (e.g. CMS database). The external state machine will then notify the smart contract when validation of the user credentials has been finished.
  2. Off-chain Signature Pattern: The off-chain signatures pattern utilizes digital signatures to validate an off-chain response. In the off-chain signatures pattern, a smart contract would access information from an external database but would require signatures from all stakeholders to be written and validated to the blockchain. For example, if a patient was trying to verify their CMS credentials, the patient information retrieving their CMS information would need to be signed by the payor, provider, and patient in order to be validated and written to the chain.

**TOKEN USE:**

**ERC-20 Token:** ERC-20 is a technical standard to implement tokens on the Ethereum blockchain. The ERC-20 standard describes a common set of functions, rules, and execution that should be followed for a token to function properly within the Ethereum ecosystem. Following the ERC-20 standard ensures tokens which are transferred within the Ethereum blockchain and how their respective supply and address balances are being consistently recorded.

**PROPOSED SYSTEM ARCHITECTURE:**

**Event Listeners:** Event Listeners are Web3 objects that are invoked upon an event being generated by the Ethereum blockchain. Event listeners are used to populate real-time information that is being written to the blockchain. View/pure Solidity contract calls are non-writing functions that merely read on-chain information and do not perform any transactional logic. This allows for information in smart contract data structures to be retrieved and displayed in the GUIs of the system’s web/mobile applications.

**DISCUSSION:**

Decision Flow Chart for use of Blockchain Technology (adopted from *Wust & Gervais*):

| **Step** | **Answer and Reasoning** |
| --- | --- |
| Do you need to store state? | Yes: All information related to healthcare claims must be stored. The current state of the adjudication process is necessary to the system. |
| Are there multiple writers? | Yes: multiple stakeholders such as payers, providers and patients will write information to storage. |
| Can you use an always online Trusted Third Party (TTP)? | No: It is impractical for any TTP (such as a government healthcare agency) of always being online to have the capability of verifying the high throughput of healthcare claims through traditional solutions. |
| Are all writers known? | Yes: All writers will be needed to register or be verified in the system. |
| Are all writers trusted? | No: Due to different incentives of stakeholders in the system, it can be beneficial to a stakeholder to be complicit in fraud for profit. |
| Is public verifiability required? | No: Verifiability is required but not in the public domain to due privacy of healthcare information. |
